# IFN-**κ** is critical for normal wound repair and is decreased in diabetic wounds

**DOI:** 10.1172/jci.insight.152765

**Published:** 2022-05-09

**Authors:** Sonya J. Wolf, Christopher O. Audu, Amrita Joshi, Aaron denDekker, William J. Melvin, Frank M. Davis, Xianying Xing, Rachael Wasikowski, Lam C. Tsoi, Steven L. Kunkel, Johann E. Gudjonsson, Mary X. O’Riordan, J. Michelle Kahlenberg, Katherine A. Gallagher

**Affiliations:** 1 Section of Vascular Surgery, Department of Surgery,; 2 Department of Dermatology,; 3 Department of Pathology,; 4 Department of Microbiology and Immunology, and; 5 Division of Rheumatology, Department of Internal Medicine, University of Michigan, Ann Arbor, Michigan, USA.

**Keywords:** Inflammation, Diabetes, Epigenetics

## Abstract

Wound repair following acute injury requires a coordinated inflammatory response. Type I IFN signaling is important for regulating the inflammatory response after skin injury. IFN-κ, a type I IFN, has recently been found to drive skin inflammation in lupus and psoriasis; however, the role of IFN-κ in the context of normal or dysregulated wound healing is unclear. Here, we show that *Ifnk* expression is upregulated in keratinocytes early after injury and is essential for normal tissue repair. Under diabetic conditions, IFN-κ was decreased in wound keratinocytes, and early inflammation was impaired. Furthermore, we found that the histone methyltransferase mixed-lineage leukemia 1 (MLL1) is upregulated early following injury and regulates *Ifnk* expression in diabetic wound keratinocytes via an H3K4me3-mediated mechanism. Using a series of in vivo studies with a geneticall y engineered mouse model (*Mll1*^fl/fl^ *K14*^cre–^) and human wound tissues from patients with T2D, we demonstrate that MLL1 controls wound keratinocyte–mediated *Ifnk* expression and that *Mll1* expression is decreased in T2D keratinocytes. Importantly, we found the administration of IFN-κ early following injury improves diabetic tissue repair through increasing early inflammation, collagen deposition, and reepithelialization. These findings have significant implications for understanding the complex role type I IFNs play in keratinocytes in normal and diabetic wound healing. Additionally, they suggest that IFN may be a viable therapeutic target to improve diabetic wound repair.

## Graphical abstract

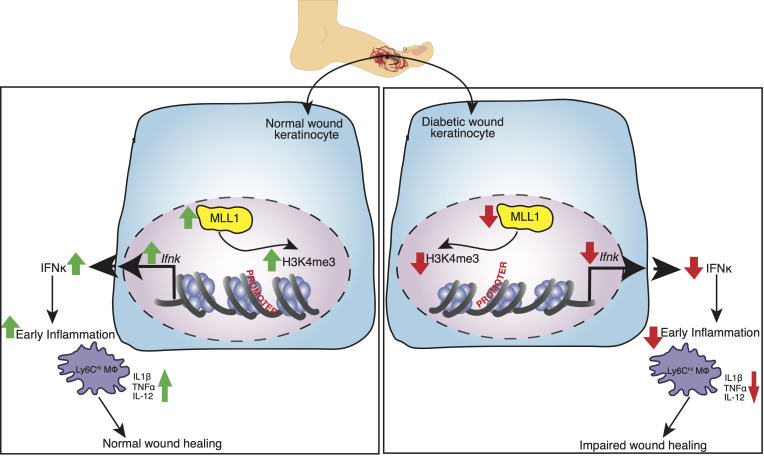

## Introduction

Impaired cutaneous wound healing is the most common complication associated with type 2 diabetes (T2D), and “standard therapy” is only marginally effective; thus, more effective treatment strategies are necessary (1, 2). Wound healing is a complex process involving the coordination of multiple cell types through the stages of homeostasis, inflammation, proliferation/reepithelization, and resolution/remodeling. Our lab and others have found that nonhealing diabetic wounds often display impaired inflammation early after injury, followed by a delayed increase in the inflammatory state (3–6). Although macrophages (MΦ) are an essential immune cell regulating wound inflammation, keratinocytes have become increasingly of interest for their part in driving skin inflammation through the production of type I IFNs (7–10); however, the role and timing of keratinocyte-mediated type I IFNs on inflammation and repair in normal and diabetic skin after injury is unknown.

The precise timing of inflammation is critically important for proper tissue repair following injury. Early after injury, there is increased expression of inflammatory cytokines (e.g., *Il6*, *Il1b*, and *Tnfa*), which are destructive to both the tissue and pathogens. This stage is vital for clearing cellular debris and invading microbes in order to facilitate tissue repair and cellular proliferation in later stages (11). Our lab has shown that early impaired expression of inflammatory cytokines in monocyte/MΦ, primarily in the dermis, impairs wound healing, and diabetic wounds fail to upregulate those cytokines early following injury (12). During wound repair, crosstalk between the epidermis and dermis can regulate inflammation (13). The epidermis is composed mainly of keratinocytes, and these cells function as the first link in cutaneous immunity through the release of key cytokines/chemokines that initiate the wound-healing cascade (13). Typically following cutaneous injury, keratinocytes secrete multiple cytokines, including IL-6 and type I IFNs (i.e., IFN-α, IFN-β, and IFN-κ) to activate and recruit immune cells to the site of injury (10, 14–17). Keratinocytes are further subdivided into keratinized, differentiated, and undifferentiated (basal) cells based on morphology and position in the epidermis (10). Basal keratinocytes are important for reepithelization during wound healing, and diabetic wound basal keratinocytes display impaired migration, proliferation, and differentiation (18–21). Data examining their role during the inflammatory phase of repair is limited; however, it is known that basal keratinocyte production of IFN-κ is important for driving skin inflammation in lupus and psoriasis (15, 16, 22, 23). Thus, keratinocyte production of IFN-κ may play a critical, previously unrecognized role in initiating early inflammatory responses that mitigate wound repair.

Epigenetic regulation plays a vital role in the phenotype and function of cells during wound healing. This process is tightly controlled within a cell and can suppress or promote the expression of specific genes important for inflammation. However, current studies of epigenetic regulation in keratinocytes have been highly limited and are relevant only to normal wound repair and commercial cell lines. For example, histone demethylase jumonji domain containing-3 (JMJD3), which removes a methyl group on the histone 3 lysine 27 (H3K27), is important for regulating migration and NF-κB regulated cytokine expression in keratinocytes during normal wound repair (24, 25). Our group has previously shown that mixed-lineage leukemia 1 (MLL1), an activating histone methyltransferase that specifically methylates lysine 4 on histone 3 (H3K4), is important for the regulation of MΦ phenotypes during wound repair and is decreased early in diabetic wound MΦ (26, 27); however, the role of MLL1 in other cell types (e.g., keratinocytes) following injury, is unknown.

Here, we demonstrate that IFN-κ is important for normal wound repair and is impaired in diabetic keratinocytes in vivo. We found that keratinocyte *Ifnk* expression was elevated early following injury, and global KO of IFN-κ (*Ifnk*^–/–^) demonstrated impaired wound healing. Furthermore, there was a decrease in expression of inflammatory cytokines (*Il1b*, *Tnfa*, *Il12*) and inflammatory MΦ early in wounds isolated from *Ifnκ*^–/–^ mice compared with controls. Next, we examined IFN-κ levels in T2D patient wounds and non-T2D control wounds, and we showed a decrease in human T2D wound keratinocytes. Furthermore, isolated keratinocytes from diabetic mice demonstrated decreased *Ifnk* expression compared with keratinocytes isolated from WT controls. Since previous work has shown that MLL1 regulates the inflammatory response in MΦ in diabetic wounds, we examined *MLL1* expression in keratinocytes from T2D and non-T2D human wounds by single-cell RNA-Seq (scRNA-Seq). We found that *MLL1* expression is impaired in keratinocytes from T2D wounds compared with controls. We then examined this in vivo in isolated keratinocytes from murine diabetic wounds and found that *Mll1* expression was decreased. ChIP analysis of H3K4me3 and MLL1 deposition at the *Ifnk* promoter in diabetic and control wound keratinocytes isolated at an early time point after injury revealed a decrease in H3K4me3 and MLL1 in diabetic keratinocytes compared with control. Using keratinocyte-specific MLL1-KO mice (*Mll1*^fll/fl^ *K14*^cre+^), generated in-house, we demonstrate that MLL1 regulates *Ifnk* expression in wound keratinocytes and that keratinocyte-specific loss of MLL1 resulted in early impaired wound healing and inflammatory cytokine expression. Finally, administration of IFN-κ early following injury improved diabetic wound repair through increasing early inflammation, collagen deposition, and reepithelialization. These findings suggest that MLL1-regulated early *Ifnk* expression in keratinocytes may be a viable therapeutic target to improve diabetic wound repair.

## Results

### IFN-κ is critical for normal wound repair.

**Figure 1:**
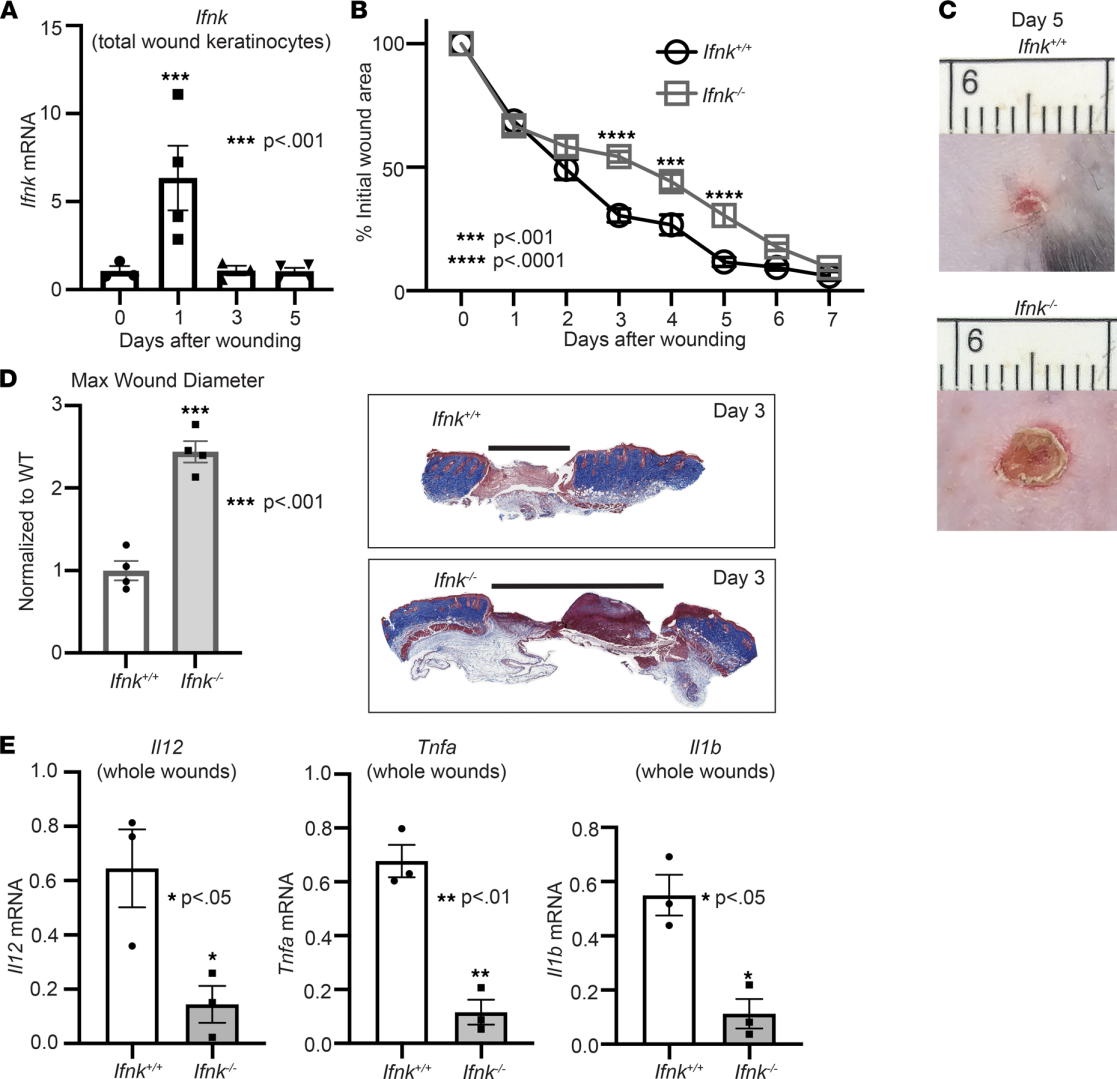
IFN-κ is important for normal tissue repair. (**A**) Total wound keratinocytes were isolated from day 0, 1, 3, and 5 wounds, and *Infk* expression was examined via qPCR. *n* = 3–5 mice per group, repeated 2 times in triplicate. (**B**) The 4 mm punch biopsy wounds were created on WT (*Ifnk*^+/+^) and IFN-κ–KO (*Ifnk*^–/–^) mice. The change in wound area was recorded daily and analyzed with ImageJ software (2 wounds per mouse, *n* = 5 mice per group). (**C**) Representative photographs of the wounds were taken on day 5. (**D**) Wound diameter at day 3 for *Ifnk*^+/+^ and *Ifnk*^–/–^; *n* = 4 per group. Trichrome staining representation is shown. (**E**) Wounds isolated from WT and IFN-κ–KO mice on day 3, *n* = 3 per group. Gene expression of inflammatory cytokines *Tnf*, *Il1b*, and *Il12* was measured via qPCR. Data were analyzed for variances, and 2-tailed Student’s *t* test or 1-way ANOVA was performed. *P < 0.05, **P < 0.01, ***P < 0.001, and ****P < 0.0001. Data are presented as mean ± SEM.

Given that IFN-κ has been shown to play a role in inflammatory skin diseases such as lupus (10), and that coordinated inflammation is important for proper wound healing, we first examined the role of IFN-κ in wound repair. In order to explore this, C57BL/6J mice were wounded with a 4 mm punch biopsy, as previously described by our group (28), and total wound keratinocytes were isolated on days 0, 1, 3, and 5 following injury (Figure 1A) (29). We found that *Ifnk* expression is upregulated by day 1 in keratinocytes, suggesting an importance for IFN-κ early following injury. To determine if loss of IFN-κ impacts normal wound repair, we utilized rederived IFN-κ–KO (*Ifnk*^–/–^) mice. These mice are globally deficient in the *Ifnk* gene. Following injury, wound closure was monitored daily using NIH ImageJ software, and the epithelial gap was measured by histology as we have previously described (30). Mice deficient in IFN-κ demonstrated significantly impaired healing compared with WT control mice (*Ifnk*^+/+^) (Figure 1, B and C, and Supplemental Figure 1; supplemental material available online with this article; https://doi.org/10.1172/jci.insight.152765DS1). Epithelial gap measurements and representative images from day 3 are shown (Figure 1D). Since IFN-κ has been shown to regulate inflammation and since early inflammation is important for normal wound repair, we examined if wounds isolated from IFN-κ–deficient mice demonstrated changes in inflammatory cytokine expression (*Il1b*, *Tnfa*, *Il12*). We specifically examined relevant cytokines known to be highly important in early wound repair. Interestingly, wounds isolated from *Ifnk*^–/–^ mice demonstrated decreased inflammatory cytokine expression by day 3 (Figure 1E). Taken together, these data indicate that IFN-κ production by keratinocytes is important for regulating early inflammation following injury, which may be necessary for normal tissue repair.

### IFN-κ is important for immune cell recruitment and regulation of MΦ phenotype in wounds.

**Figure 2:**
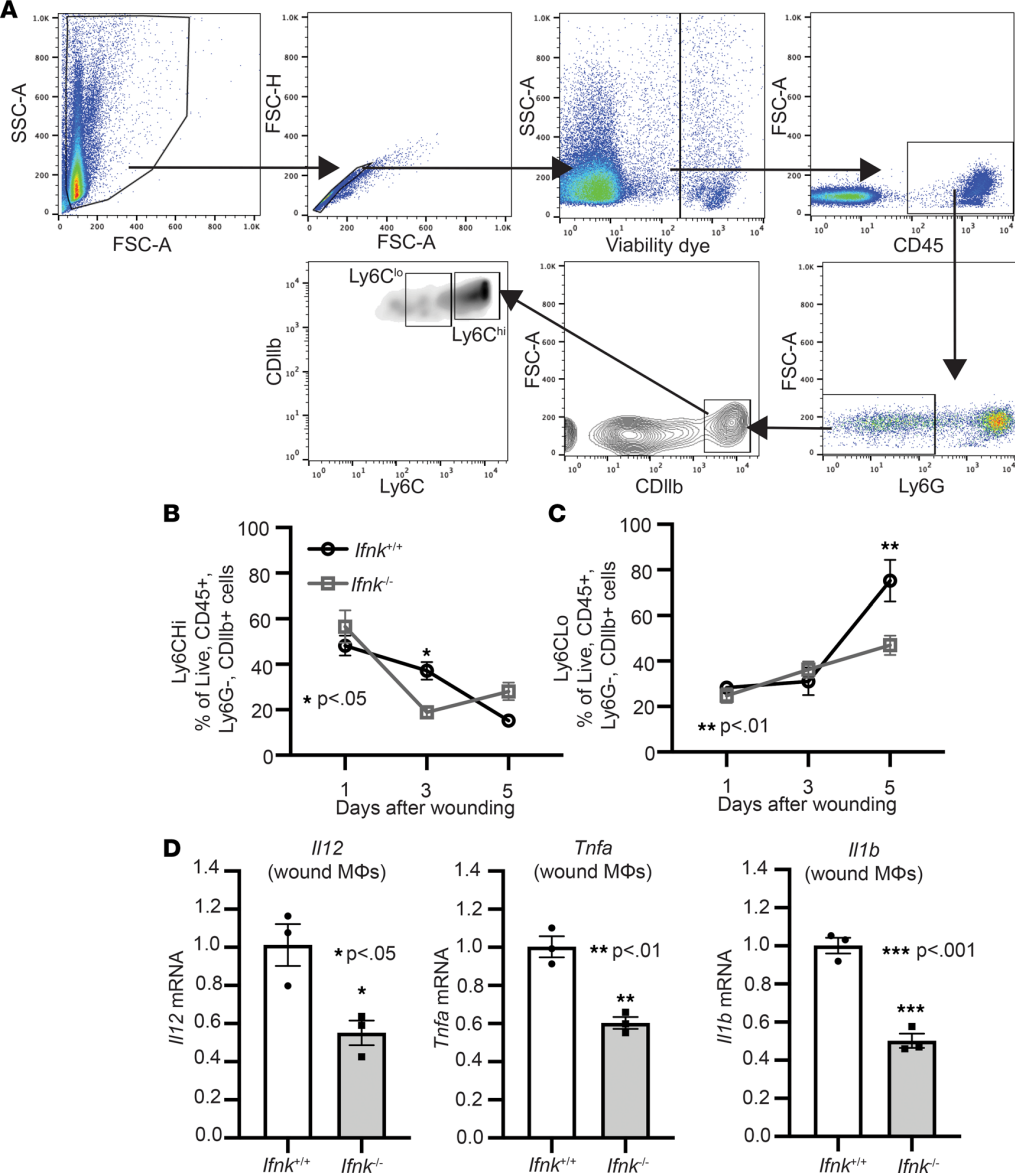
IFN-κ regulates macrophage inflammatory profile during normal tissue repair. (**A**) IFN-κ–KO and control wound cell isolates were processed for flow cytometry using the following gating strategy selecting for single cells, live (viability dye^–^), CD45^+^, Ly6G^−^, CD11b^+^, Ly6C^hi^, or Ly6C^lo^. (**B** and **C**) Flow cytometry quantification of Ly6C^hi^ and Ly6C^lo^ cells in wounds (*n* = 4–6 per group). (**D**) Wound monocyte/macrophages (MΦ) (CD3^–^CD19^–^Ly6G^–^CD11b^+^) were isolated from WT and IFN-κ–KO mice on day 3; *n* = 3 per group, repeated in triplicate. Gene expression of inflammatory cytokines *Tnf*, *Il1b*, and *Il12* was measured via qPCR. Data were analyzed for variances, and 2-tailed Student’s *t* test or 1-way ANOVA was performed. *P < 0.05, **P < 0.01, and ***P < 0.001. Data are presented as mean ± SEM.

Immune cell recruitment is a hallmark during wound healing. In particular, the dichotomous roles of monocytes/MΦ are important for normal tissue repair (12, 31, 32). Our lab has shown that Ly6C^hi^ monocytes/MΦ display an inflammatory profile and are increased early after injury. In contrast, Ly6C^lo^ monocytes/MΦ exhibit an antiinflammatory profile and are increase later after injury (12). Given that we see a decrease in monocyte/MΦ-associated inflammatory cytokine expression early in whole wounds, we examined changes in the Ly6C^hi^ and Ly6C^lo^ populations in *Ifnk*^–/–^ mice and control mice following injury. Using the gating strategy in Figure 2A, we see a significant decrease in Ly6C^hi^ monocytes/MΦ in *Ifnk*^–/–^ mice compared with control on day 3 after injury (Figure 2B). This early impairment was followed by a late increase in Ly6C^hi^ monocyte/MΦ by day 5. Compared with their counterparts, Ly6C^lo^ cells are decreased on day 5 after injury in *Ifnk*^–/–^ mice compared with control (Figure 2C). To evaluate if wound monocytes/MΦ displayed changes in inflammatory cytokine expression early following injury in *Ifnk*^–/–^ and WT mice, CDllb^+^ monocytes/MΦ were isolated from wounds on day 3 and were examined for *Il12*, *Tnfa*, and *Il1b* expression. Comparably, a significant decrease in *Il12*, *Tnfa*, and *Il1b* was noted in CDllb^+^ monocytes/MΦ from *Ifnk*^–/–^ mice (Figure 2D). Based on these data, IFN-κ contributes to monocyte/MΦ recruitment and inflammatory phenotype in wounds. Given that neutrophils and T cells also play a role in wound healing, we examined changes in these cell populations. While we did not see a significant difference in neutrophil recruitment, we did see a significant increase in T cells on day 3 in wounds from *Ifnk*^–/–^ mice compared with control (Supplemental Figure 2). These findings suggest that IFN-κ plays a part in immune cell recruitment and MΦ phenotype during wound healing.

### IFN-κ is decreased in human and murine diabetic wounds.

**Figure 3:**
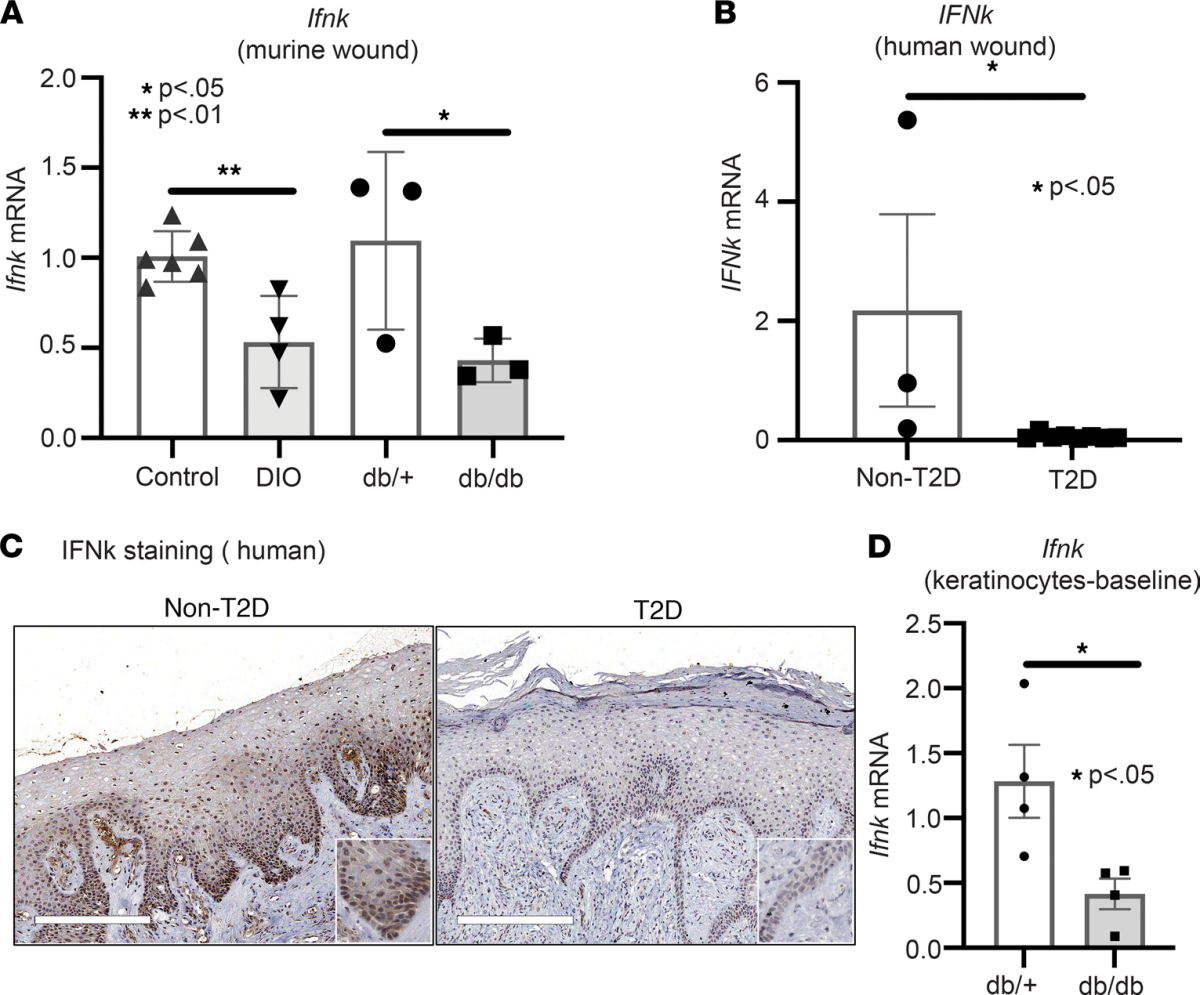
Impaired IFN-κ production in human diabetic wounds. (**A**) Whole wounds were isolated from 2 diabetic models (DIO and *db/db*) and their respective controls; *n* = 3–6 per group. *Ifnk* gene expression was measured via qPCR. (**B**) Human wounds were collected from T2D patients with chronic diabetic foot ulcers and non-T2D; *n* = 3–8 per group. *IFNk* gene expression was measured via qPCR. (**C**) Representative staining for IFN-κ performed on wounds from T2D and non-T2D patient skin. Magnification, ×10. (**D**) Keratinocytes were isolated at baseline from the tail of *db/db* mice and their controls; *n* = 4 per group, repeated in triplicate. *Ifnk* expression was measured via qPCR. Data were analyzed for variances, and 2-tailed Student’s *t* test or Mann-Whitney *U* test was performed. *P < 0.05 and **P < 0.01. Data are presented as mean ± SEM.

Given that we identified that *Ifnk* expression is increased early following injury in keratinocytes and is important for regulating inflammation during wound healing, we next examined if there were differences in *Ifnk* expression in diabetic wounds compared with controls early after injury. In order to study physiologic T2D, we used the diet-induced obesity (DIO) mice, which are C57BL/6J mice placed on a high-fat diet (60% saturated fat) for 12–16 weeks. We found impaired *Ifnk* expression in DIO wounds compared with controls. We confirmed this finding in a second murine model of T2D, the *db/db* mice, which lack a functional leptin receptor (Figure 3A) (33). To see if this was translatable to human T2D patients, we examined *IFN-k* expression from human wounds isolated from T2D patients with chronic diabetic foot ulcers and non-T2D controls. Similarly, T2D wounds revealed attenuated *IFN-k* expression in diabetic wound tissue compared with non-T2D wounds (Figure 3B). In order to examine if these *IFN-k* gene expression changes translated into changes at the protein level, we performed IHC with antibodies to IFN-κ. This staining identified that IFN-κ protein was reduced in human T2D wounds compared with non-T2D controls (Figure 3C). In particular, this decrease was seen most prominently in the epidermal basal keratinocytes, with some expression also noted cells in the dermis. Since we found IFN-κ to be impaired in human and murine diabetic wounds, we wanted to examine expression in keratinocytes at baseline. Thus, we isolated keratinocytes from *db/db* mice and their control, and following culture for 3 days, we examined *Ifnk* expression via quantitative PCR (qPCR). Additionally, we identified that *Ifnk* expression is significantly impaired in *db/db* keratinocytes compared with controls at baseline (Figure 3D). These results indicate that IFN-κ is decreased in diabetic keratinocytes and may contribute to the early impaired inflammation. 

### IFN-κ expression in diabetic wound keratinocytes is epigenetically regulated by MLL1.

**Figure 4:**
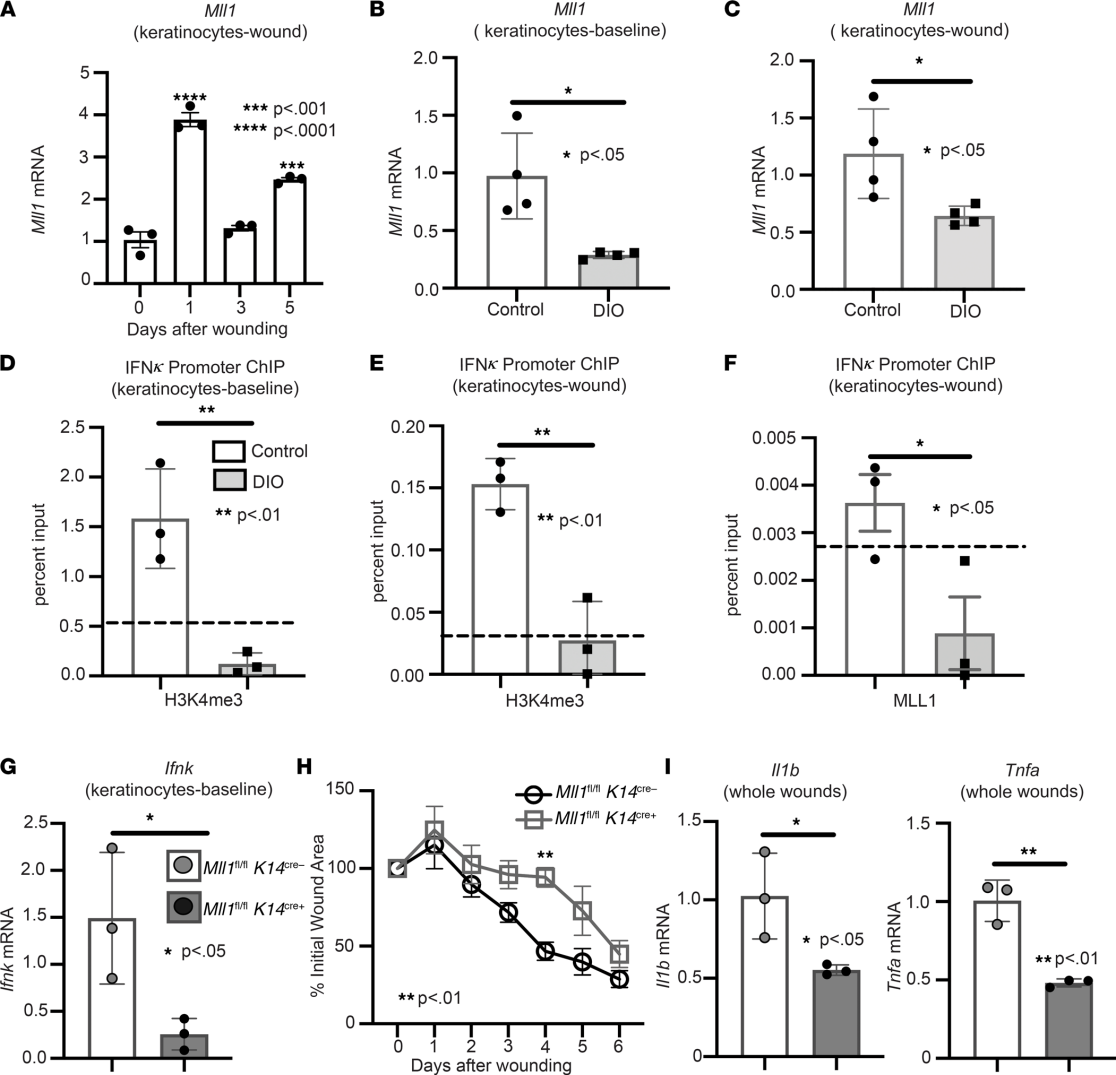
IFN-κ expression in diabetic keratinocytes is regulated by MLL1. (**A**) Wound keratinocytes were isolated from day 0, 1, 3, and 5 wounds. *Mll1* expression was examined via qPCR. *n* = 3 mice per group repeated 2 times in triplicate. (**B** and **C**) Keratinocytes at baseline and wounds were isolated from ND and DIO mice. *Mll1* gene expression was measured via qPCR (*n* = 4 mice per group, repeated in triplicate). (**D** and **E**) Keratinocytes at baseline and wounds were isolated from DIO and control mice. ChIP analysis for H3K4me3 deposition at the *Ifnk* promoter was performed in keratinocytes from wounds and at baseline of DIO and control mice (*n* = 3 per group, repeated in triplicate). Respective IgG controls (dotted line). (**F**) Keratinocytes were isolated from DIO and control wounds. ChIP analysis for MLL1 deposition at the *Ifnk* promoter was performed (*n* = 3 per group, repeated in triplicate). The dotted line indicates the respective IgG controls. (**G**) Keratinocytes were isolated from *Mll1*^fl/fl^ *K14*^cre+^ and littermate controls and analyzed for *Ifnk* expression by qPCR (*n* = 3 mice per group, repeated in triplicate). (**H**) The 4 mm punch biopsy wounds were created on *Mll1*^fl/fl^ *K14*^cre+^ and littermate controls. The change in wound area was analyzed with ImageJ software (2 wounds per mouse, *n* = 4–5 mice per group). (**I**) Wounds isolated from *Mll1*^fl/fl^ *K14*^cre+^ and control mice on day 3; *n* = 3 per group. Gene expression of inflammatory cytokines *Tnf* and *Il1b* were measured via qPCR. Data were analyzed for variances, and 2-tailed Student’s *t* test or 1-way ANOVA was performed. *P < 0.05, **P < 0.01, ***P < 0.001, and ****P < 0.0001. Data are presented as mean ± SEM.

We and others have shown MLL1, an activating histone methyltransferase that specifically methylates H3K4, is important in MΦ for wound healing (34–37); however, whether MLL1 regulates keratinocyte gene expression following injury remains unknown. To examine the kinetics of *Mll1* expression during wound healing, C57BL/6J mice were subjected to 4 mm full-thickness wounds as previously described (28), and keratinocytes were isolated from wounds at multiple time points (0, 1, 3, and 5 days) and analyzed for *Mll1* expression. We found that *Mll1* had the highest upregulation at day 1 after injury (Figure 4A), which correlates with the timing of *Ifnk* increased expression during normal wound healing. Since MLL1 has been shown by our group to be decreased at early time points in diabetic wounds, and since we found that *Ifnk* expression is reduced in diabetic keratinocytes early following injury, we examined whether *Ifnk* expression in diabetic keratinocytes was epigenetically regulated by MLL1. We isolated keratinocytes from wounds and at baseline and found that *Mll1* expression was impaired in DIO keratinocytes compared with controls (Figure 4, B and C). Since MLL1 regulates gene transcription via H3K4me3, we examined H3K4me3 at the *Ifnk* promoter. We found decreased H3K4me3 on the *Ifnk* promoter in diabetic keratinocytes at baseline compared with controls (Figure 4D); this was also seen in keratinocytes isolated from diabetic wounds (Figure 4E). Additionally, we showed that MLL1 colocalization was decreased at the *Ifnk* promoter in keratinocytes isolated from diabetic wounds compared with control (Figure 4F). These data suggest that MLL1-dependent H3K4me3 deposition regulates *Ifnk* expression in keratinocytes both at baseline and following injury. To further evaluate the ability of MLL1 to regulate *Ifnk* expression in wound keratinocytes, we generated mice deficient in MLL1 in keratinocytes by using the Cre-lox system. Keratinocyte deletion of MLL1 was confirmed in vivo by examining baseline primary keratinocytes isolated from *Mll1*^fl/fl^ *K14*^cre+^ mice and littermate controls (*Mll1*^fl/fl^ *K14*^cre–^). To identify if decreased MLL1 in keratinocytes altered *Ifnk* expression, primary keratinocytes were isolated from *Mll1*^fl/fl^ *K14*^cre+^ mice and littermate controls. Keratinocytes from *Mll1*^fl/fl^ *K14*^cre+^ exhibited decreased *Ifnk* expression compared with controls (Figure 4G). These data indicate that MLL1 may control *Ifnk* expression in keratinocytes and be partially responsible for the decrease in *Ifnk* seen in diabetic keratinocytes. Given that we see a decrease in *Ifnk* expression in *Mll1*^fl/fl^ *K14*^cre+^ mice, we examined if these mice exhibited changes in wound healing and inflammatory cytokine expression. Following injury, wound closure was monitored using ImageJ software, and *Mll1*^fl/fl^ *K14*^cre+^ mice compared with controls displayed significantly impaired wound healing on day 4 (Figure 4H). *Mll1*^fl/fl^ *K14*^cre+^ mice also displayed decreased inflammatory cytokine expression of *Il1b* and *Tnfa* in wounds on day 3 after injury compared with littermate controls (Figure 4I). These data suggest that MLL1 may play a role in the impaired wound healing and inflammatory cytokine expression early after injury, in part through the regulation of *Ifnk* gene expression in keratinocytes.

### Human single-cell sequencing of T2D wounds reveals decreased MLL1 in keratinocytes.

**Figure 5:**
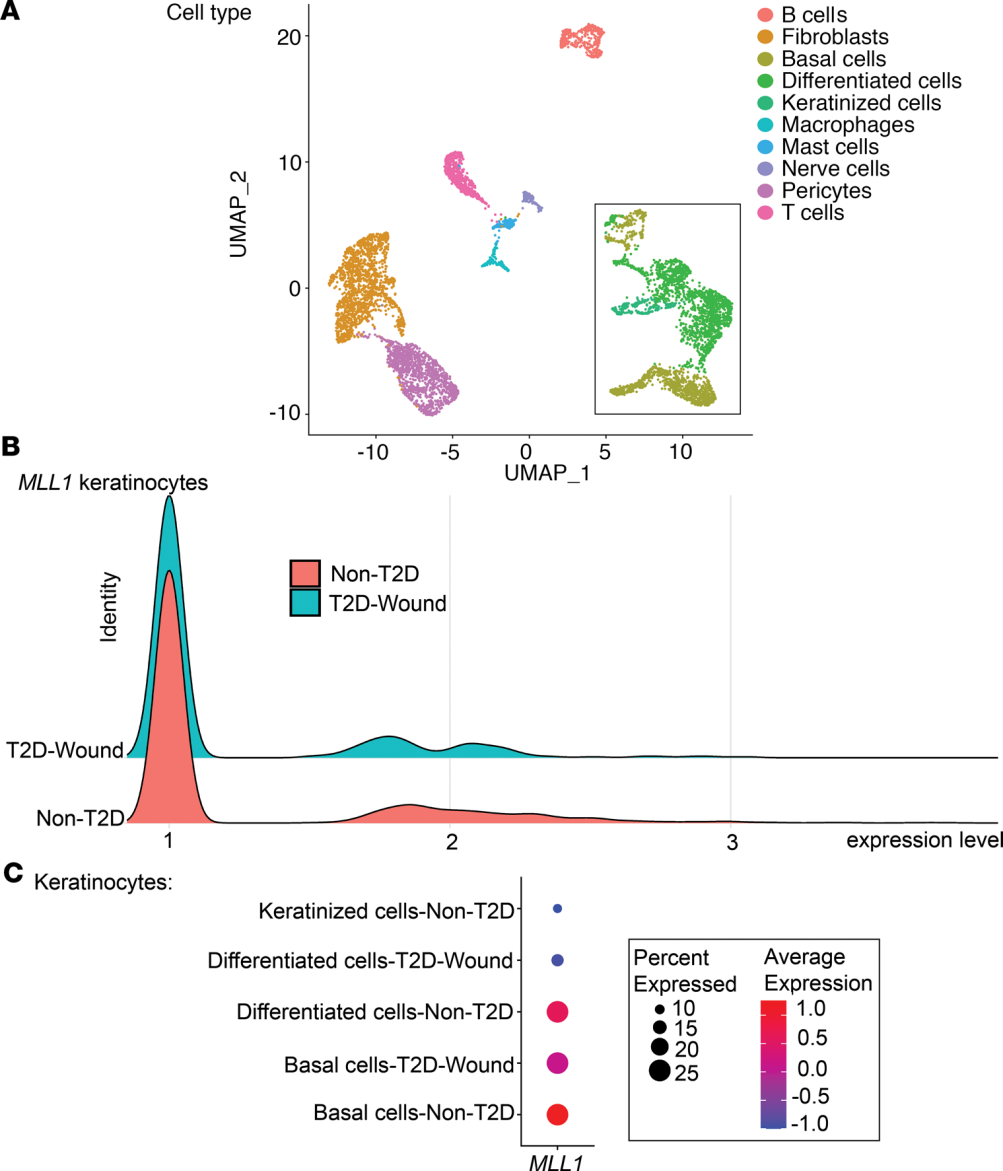
scRNA-Seq of Mll1 expression is decreased in human T2D wounds. (**A**) Cluster analysis was performed using the UMAP technique of single-cell sequencingT2D (*n* = 1) and nondiabetic wound (*n* = 2) samples. The black box outlines the keratinocyte populations: basal cells, differentiated cells, keratinized cells. (**B**) Ridge plot demonstrating expression levels of *MLL1* within keratinocytes in human T2D and non-T2D samples. (**C**) Dot plot demonstrating *MLL1* expression within keratinocyte populations in human T2D and non-T2D samples. Dot size corresponds to the proportion of cells within the group expressing *MLL1*, and dot color corresponds to expression level.

In order to examine if *MLL1* expression is decreased in human T2D keratinocytes, we performed scRNA-Seq analysis on wounds from T2D patients and non-T2D controls. Cluster analysis was performed using uniform manifold approximation and projection (UMAP) as previously described (28); this technique identified 10 cell clusters in the wound area following injury. There were 3 clusters of keratinocytes identified in the wound area, including keratinized, differentiated, and basal keratinocytes (Figure 5A). Analysis revealed that *MLL1* expression was decreased in total keratinocytes from human T2D wounds in comparison with non-T2D controls (Figure 5B); in particular, *MLL1* expression was downregulated in both basal and differentiated keratinocytes of T2D patients (Figure 5C). Although MLL1 likely regulates multiple genes in keratinocytes, these data indicate that *MLL1* is decreased in human T2D keratinocytes and may partially regulate *IFN-k* gene expression in diabetic keratinocytes.

### Administration of IFN-κ improves diabetic wound healing.

**Figure 6:**
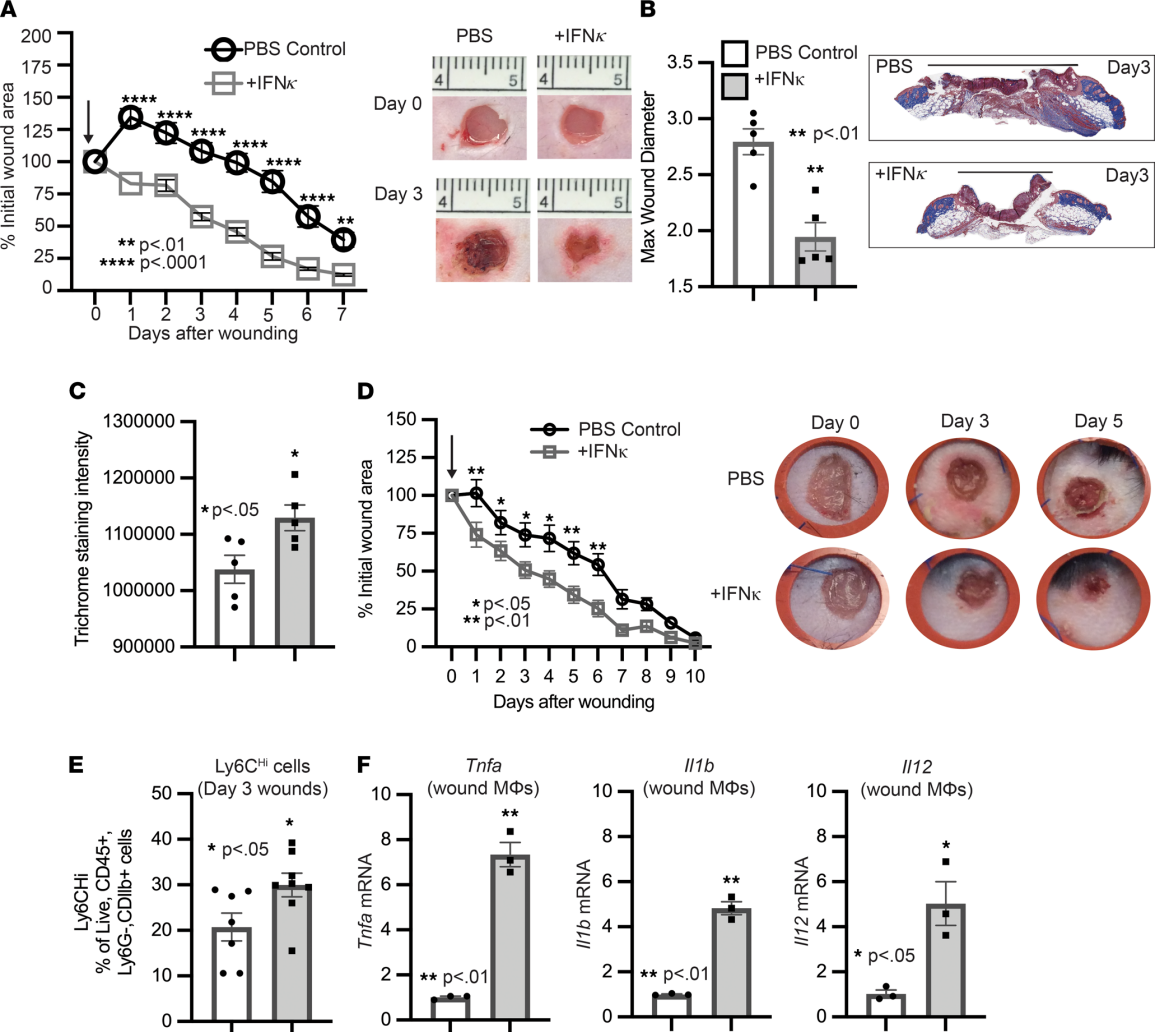
Administration of IFN-κ improves diabetic wound healing. (**A**) The 4 mm punch biopsy wounds were created on DIO, and wounds were injected daily starting on day 0 for 3 days after injury with IFN-κ (0.5 μg/100 μL) or PBS control (2 wounds per mouse, *n* = 5 mice per group, repeated once). The arrow indicates when injections started. The change in wound area was recorded daily and analyzed with ImageJ software. Representative photographs of the wounds were taken on days 0 and 3. (**B**) Max wound diameter at day 3 for IFN-κ and PBS control; *n* = 5 per group. Trichrome staining representative picture. (**C**) Trichrome staining was calculated using ImageJ software (*n* = 5 mice/group). (**D**) The 4 mm punch biopsy wounds were created and splinted on DIO mice, and wounds were injected starting on day 0 for 3 days after injury with IFN-κ (0.5 μg/100 μL) or PBS control (1 wound per mouse, *n* = 7–8 mice per group). The arrow indicates when injections started. The change in wound area was recorded daily and analyzed with ImageJ software. Representative photographs of the wounds were taken on days 0, 3, and 5. (**E**) Flow cytometry quantification of Ly6C^hi^ cells in wounds (*n* = 7–8 per group). (**F**) Wound monocyte/macrophages (MΦ) (CD3^–^CD19^–^NK1.1^–^Ly6G^–^CD11b^+^) were isolated from IFN-κ or PBS control mice on day 3; *n* = 3 per group, repeated in triplicate. Gene expression of inflammatory cytokines *Tnf*, *Il1b*, and *Il12* was measured via qPCR. Data were analyzed for variances, and 2-tailed Student’s *t* test or 1-way ANOVA was performed. *P < 0.05, **P < 0.01, and **** *P* < 0.0001. Data are presented as mean ± SEM.

Given the critical local role of IFN-κ for early initiation of inflammation for proper wound healing and its impairment in diabetic wounds, we assessed if early administration of IFN-κ locally improved wound healing in DIO mice. To examine this, DIO mice were wounded with a 4 mm punch biopsy, and IFN-κ or PBS (control) were administered daily for 3 days via local s.c. injection. Wound healing was assessed using ImageJ software, and the epithelial gap was measured by histology, as previously described (30). DIO mice treated with IFN-κ demonstrated a significant improvement in wound healing on days 1–7 (Figure 6, A and B). To understand how IFN-κ injection improves diabetic wound healing, we first examined changes in collagen deposition and angiogenesis. Histological examination using ImageJ software to measure trichrome straining intensity showed a moderate increase in collagen deposition following local injection of IFN-κ after injury in DIO mice compared with control (Figure 6C). This change was also exhibited in *Col1* mRNA levels in whole wounds isolated from IFN-κ–injected DIO mice compared with control (Supplemental Figure 3A). IFN-κ injection also increased proangiogenic factor *Vegfa* expression in whole wounds after injury in DIO mice (Supplemental Figure 3B), suggesting a role for IFN-κ in inducing angiogenesis. Given the decrease in the epithelial gap measurement following injuring in IFN-injected DIO mice and the significant initial decrease in the percent wound area, we wanted to confirm that this was not due to wound contraction and, rather, reepithelization. To test this, we used a well-established model of chronic (splinted) wound healing (28). Changes in wound area were analyzed using NIH ImageJ software. The chronic (splinted) wound model also displayed significantly improved wound healing following local injection of IFN-κ in DIO mice (Figure 6D), suggesting that IFN-κ increased reepithelization following injury. Since our lab has previously shown that Ly6C^hi^ cells are impaired early in diabetic wounds and that IFN-κ contributes in part to MΦ recruitment, we examined the effect of IFN-κ local injection on the presence of Ly6C^hi^ cells following injury in DIO mice. Interestingly, we see a significant increase in Ly6C^hi^ cells by day 3 after injury following IFN-κ injection (Figure 6E). This is comparable with an increase in inflammatory cytokine expression of *Tnfa*, *Il1b*, and *Il12* in CDllb^+^ monocytes/MΦ isolated from wounds on day 3 in IFN-κ–injected DIO mice (Figure 6F). These data suggest that IFN-κ local injection can increase the presence of inflammatory MΦ early in diabetic wounds for proper wound healing. Additionally, an increase in Ly6C^lo^ monocytes/MΦ was exhibited by day 5 in diabetic wounds following local injection of IFN-κ (Supplemental Figure 4). These results suggest that local IFN-κ administration early following injury may represent a novel therapeutic strategy for improving healing in diabetic wounds through several different mechanisms.

## Discussion

**Figure 7:**
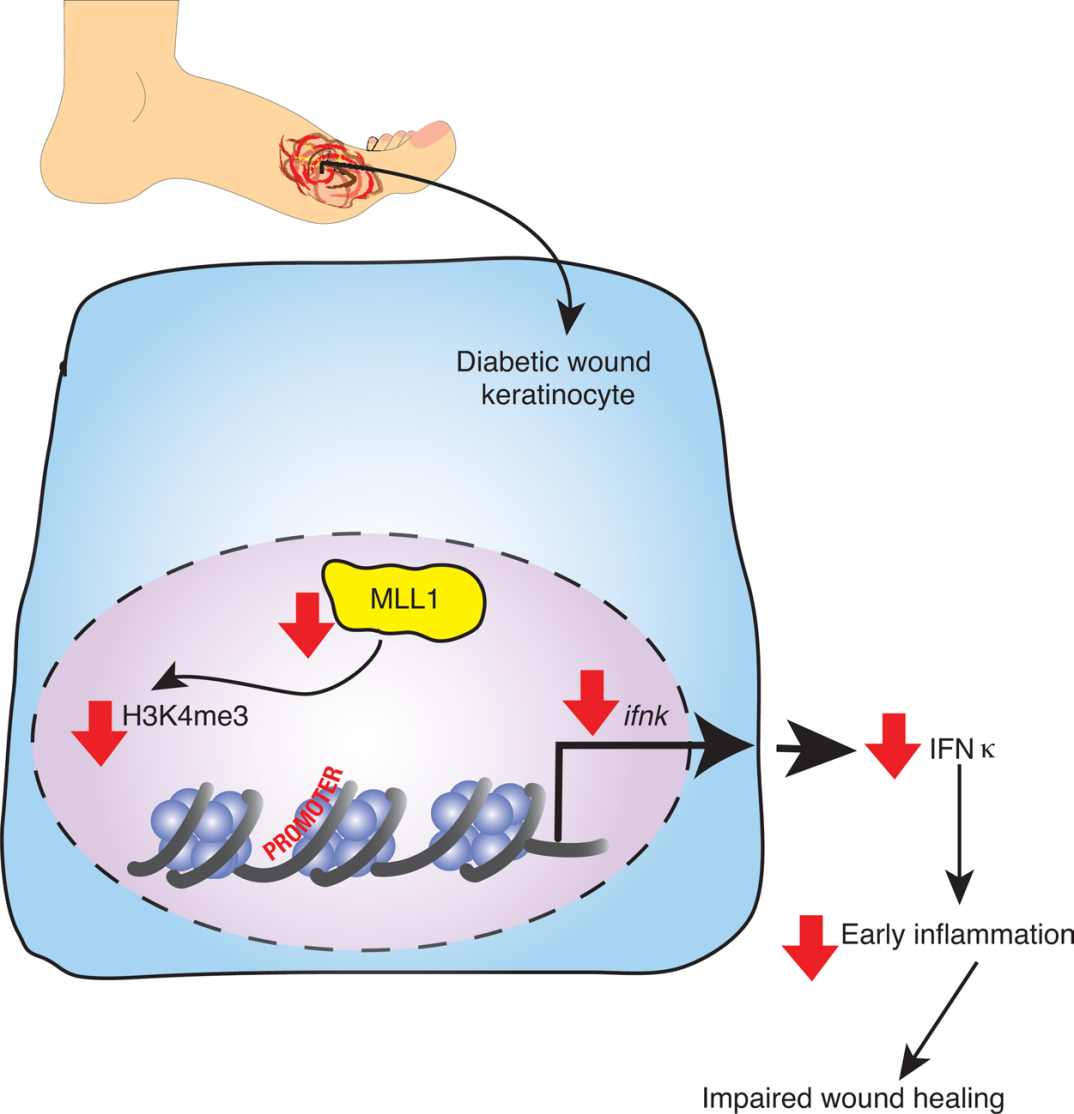
Schematic of IFN-κ regulation in diabetic wound keratinocytes.

Current literature demonstrates that an uncoordinated inflammatory response contributes to nonhealing wounds in T2D patients and that type I IFN signaling may play a role in regulating wound inflammation (6, 12). IFN-κ is a type I IFN that is mainly produced by keratinocytes and is important in regulating skin inflammation in the context of inflammatory skin diseases; however, the role of IFN-κ in the context of normal and diabetic wound healing remains unclear. In this study, we found that *Ifnk* expression is elevated early in wound healing and that loss of IFN-κ using IFN-κ–KO mice impairs wound healing and inflammation. Additionally, we found that loss of IFN-κ leads to decreased inflammatory MΦ early following injury. Importantly, we discovered that wound diabetic keratinocytes predominately displayed decreased IFN-κ expression. Next, we identified that MlL1, a histone methyltransferase, is also elevated early in wound healing and regulates *Ifnk* gene expression in keratinocytes. Utilizing human single-cell sequencing of T2D and non-T2D wounds, we identified decreased *MLL1* expression in T2D keratinocytes compared with non-T2D (control) keratinocytes. Additionally, a keratinocyte-specific KO of MLL1 (*Mll1*^fll/fl^ *K14*^cre+^) reduced *Ifnk* expression in primary keratinocytes and impaired early wound healing and inflammation. Significantly, early administration of local IFN-κ into wounds improved diabetic wound healing through several mechanisms and, thus, may provide a therapeutic target to improve diabetic wound repair. Taken together, these results suggest that IFN-κ plays a crucial role in the regulation of keratinocyte-mediated inflammation early in wound healing and that MLL1 controls IFN-κ expression in wound keratinocytes (Figure 7).

For the first time, we demonstrate that IFN-κ is important early in keratinocytes for normal wound repair, and loss of IFN-κ impairs wound healing and early inflammation. Although IFN-κ can be expressed by DCs and monocytes (38, 39), we and others found that IFN is mainly produced by keratinocytes in the skin (16, 40). Furthermore, IFN-κ expression is decreased in diabetic keratinocytes. This IFN-κ production during normal wound healing may regulate keratinocyte inflammatory cytokines (i.e., IFN-β, IFN-α, IL-6) secretion through a positive feedforward loop, which has been demonstrated in other chronic skin diseases, such as lupus and psoriasis (15, 16). Keratinocytes are also responsible for the reepithelialization of wounds following injury, and our lab has shown that type I IFNs are important for this process to occur (6). Further reepithelization in diabetic wounds is impaired, and our data suggest that restoration of IFN-κ can improve this process in diabetic wounds; however, whether this is through increased proliferation or migration is still unclear.

In addition to IFN-κ affecting keratinocytes after injury, type I IFNs have played a role on infiltrating immune cells during the inflammation phase of wound healing. Specifically, our lab has shown that type I IFN signaling is important for regulating MΦ phenotype during wound healing (6). Mainly, IFN-β (a type I IFN) is important for the MΦ switch from pro- to antiinflammatory phenotype during wound healing (6). Furthermore, the local rescue of IFN-κ in DIO mice leads to an increased presence of inflammatory MΦ and inflammatory cytokine expression (*Il1b*, *Il12*, *Tnfa*) early during wounding; however, whether this effect is direct or indirect is unclear. Other infiltrating immune cells important for normal wound healing include neutrophils and T cells. While the loss of IFN-κ didn’t seem to change neutrophil recruitment significantly, it affected T cell recruitment early following injury. We did not specifically investigate differences in T cell phenotype and mechanism; thus, this is a limitation of our study.

In addition to influencing the early inflammation phase of wound healing, our data suggest IFN-κ may influence events in the later phase of wound healing; specifically, during the proliferative and remodeling phase, fibroblasts are important for the secretion of collagen to form granulation tissue in the wound (41). Our lab has shown that this process is impaired in diabetic wounds (30), and local rescue of IFN-κ early in diabetic wounds results in increased collagen deposition. The increase in antiinflammatory MΦ exhibited later during wound healing in DIO mice injected with IFN-κ and the role these cells play in stimulating fibroblast collagen deposition suggest one possible mechanism by which IFN-κ may work. Rescue of IFN-κ early during wound healing also increases vascular endothelial growth factor (VEGF) expression after injury. Given that VEGF is a major regulator of angiogenesis (42), this suggests that rescue of IFN-κ may increase angiogenesis in diabetic wounds through regulation of VEGF.

Although this study provides insight into the mechanisms behind impaired inflammation in diabetic wound healing, some limitations must be addressed. First, even though keratinocytes mainly express IFN-κ in the skin, other immune cells such as MΦ and DCs express low levels of IFN-κ and may be partially contributing to the type I IFN responses in the wound; thus, further studies are warranted examining each of these cell types. Furthermore, although MLL1 appears to influence *Ifnk* expression in wound keratinocytes, other epigenetic enzymes important in wound repair may also regulate keratinocyte *Ifnk* expression. Given that we also see an increase in *Mll1* expression on day 5 following injury, MLL1 may also influence other pathways in keratinocytes. Additionally, although local administration of IFN-κ improved wound healing and increased early inflammation, this effect could be through direct or indirect stimulation of keratinocytes or other immune and structural cells involved in wound repair.

To our knowledge, this study is the first to examine the role of IFN-κ in keratinocytes in both normal and diabetic wound healing. We have established that IFN-κ is upregulated early in keratinocytes after injury and is crucial for normal healing. Loss of IFN-κ results in an early impaired inflammatory response, which alters wound resolution. Interestingly, expression of IFN-κ is impaired in keratinocytes from T2D patients and mice compared with control. Importantly, we identified that an epigenetic methyltransferase, MLL1, could regulate *Ifnk* gene expression in keratinocytes. Furthermore, local administration of IFN-κ early following injury improved diabetic wound closure, in part through modifying early inflammation. Thus, IFN-κ and or MLL1 may serve as a therapeutic target to enhance wound healing in diabetes. Although the exact upstream mechanisms regulating MLL1-mediated *Ifnk* gene expression in keratinocytes remains vague, understanding how MLL1/IFN-κ may contribute to inflammation in wounds will enhance knowledge regarding its role in inflammation in diabetic wounds.

## Methods

### Study design.

For the murine models, we used 2 diabetic models, DIO and *db/db*, which were used in acute wound models. Before these experiments, hyperglycemia was confirmed via glucose tolerance testing. Female C57BL/6J mice do not develop glucose intolerance/insulin resistance metabolic syndrome or “prediabetes” on a high-fat diet; therefore, they cannot be used in this T2D model (43, 44). For *db/db* mice, both male and female mice were used in experiments. Studies in the genetically engineered murine model of *Mll1*^fl/fl^ *K14*^cre+^ used a minimum of 3–5 mice per group. The number of mice per group to ensure sufficient sample sizes for all experiments was determined based on prior literature and experience from our previous studies. No mice were excluded from analysis. Details on experimental replicates are indicated in figure legends.

### Mice.

All mice were housed at University of Michigan animal facilities. C57BL/6J, *db/db*, *db/+*, K14^cre+^ mice were purchased from The Jackson Laboratory at 6–7 weeks of age and maintained in breeding pairs in Unit for Laboratory Animal Medicine (ULAM) facilities. *Ifnk*^–/–^ mice on a C57BL/6J background were a gift from M. Kahlenberg (University of Michigan) (23). *Mll1*^fl/fl^ mice on a C57BL/6J background were obtained from T. Billiar (University of Pittsburgh, Pittsburgh, Pennsylvania, USA). Mice with the *Mll1* gene deleted in keratinocytes were generated by mating *Mll1*^fll/fl^ mice with *K14*^cre+^ mice. All mice were housed on a 14-hour light/10-hour dark cycle with free access to food, water, and bedding (Andersons Lab Bedding Bed-o’Cobs combination).

To induce a “diabetic” state, male C57BL/6J mice were maintained on a high-fat diet (60% kcal fat; Research Diets) or a normal chow diet (13.5% kcal fat; LabDiet) for 12–16 weeks, to generate the DIO model, which mirrors human physiology in dietary-induced weight gain and the development of insulin resistance and glucose intolerance (3–5). To confirm that our findings were not specific to the DIO murine model, we also used the *db/db* murine model.

### Wound-healing assessment.

For the acute wound-healing model, mice were anesthetized with isoflurane using a vaporizer with the station flowmeter set at 2.0 liters per minute (LPM). Mice were depleted of hair on their dorsum via Veet (Reckitt Benckiser), and two 4 mm punch biopsy wounds were created per mouse, as previously described (27). For the chronic wound-healing model, a splinted full-thickness wound was used to minimize wound contracture as previously described (28). Briefly, a 4 mm full-thickness excisional wound was created on the shaved dorsum of the anesthetized mice. A 10 mm doughnut-shaped silicone splint was then centered on the wound and fixed to the skin using interrupted 6-0 nylon sutures (Ethicon). In IFN-κ rescue experiments, 0.5 μg IFN-κ (R&D Systems, 8437-MK) or 100 μL PBS control injection was performed s.c. at 2 points along the wound edge, as described previously by our group (28). For the acute and chronic wound-healing model, an iPad camera (8-megapixel) was used to take digital photographs daily. The wound surface area was calculated using ImageJ software (NIH) and was expressed as percentage of original wound size/ time. The acute wounds were collected at the indicated time points and immediately snap-frozen and placed at –80°C for RNA isolation, prepared for keratinocyte isolation, placed in formalin for histology, or processed for flow cytometry. Day 0 wounds were collected 2 hours after injury. For keratinocyte isolation experiments, 6 mm wound biopsies were obtained, encompassing a 2 mm margin around the wound.

Some acute wounds were harvested and formalin fixed overnight, followed by embedding the tissue in paraffin. Murine tissue slides were stained with Masson’s trichrome stain and H&E, as previously described (30). The wound diameter was obtained by calculating the distance between leading wound epithelial edges compared with the maximum diameter of the wound. Images were taken using a Zeiss Axioskop 2 microscope. Trichrome staining intensity was calculated using ImageJ software.

### Human wound isolation.

Wounds were isolated from T2D patients with chronic wounds (greater than 2 months) and non-T2D patients who were undergoing amputation. An 8 mm full-thickness punch biopsy was obtained immediately and processed from discarded skin. Paraffin-embedded tissue slides obtained from T2D patients and control were stained for IFN-κ (16). Slides were heated for 30 minutes at 60°C. This was followed by rehydration and epitope retrieval with Tris-EDTA (pH 9). Slides were then blocked and incubated with primary antibody (IFN-κ, Abnova, H00056832-M01; mouse IgG2a, κ isotype control, Thermo Fisher Scientific, 14-4724-81) overnight at 4°C.

For scRNA-Seq, 8 mm punch biopsies from diabetic wounds and normal skin samples were collected. In the diabetic patient cohort, the average age was 60 years. All patients exhibited diabetes, hyperlipidemia, hypertension, and coronary artery disease. In the nondiabetic patient cohort, the average age was 70 years, with half of the patients having hyperlipidemia, hypertension, and coronary artery disease. scRNA-Seq was done as previously described (28). scRNA-Seq cell suspension was performed as previously published (28). Skin was harvested with punch biopsy from diabetic and nondiabetic control patient wounds. Samples were then incubated in 0.4% dispase (Thermo Fisher Scientific) in HBSS (Thermo Fisher Scientific) at 4°C overnight. Following separation of the epidermis and dermis, the epidermis was digested in 0.25% Trypsin-EDTA (Thermo Fisher Scientific) with 10 units/mL DNase I (Thermo Fisher Scientific) at 37°C for 1 hour. This reaction was quenched with FBS (Atlanta Biologicals) and passed through a 70 μM mesh. The dermis was minced and incubated in 0.2% Collagenase II (Thermo Fisher Scientific) with 0.2% Collagenase V (MilliporeSigma) in a plain medium for 1.5 hours at 37°C and passed through a 70 μM mesh. Dermal and epidermal cells were combined in a 1:1 ratio for scRNA-Seq, performed by the University of Michigan Advanced Genomics Core on the 10x Genomics Chromium System. Libraries were sequenced on the Illumina NovaSeq 6000 sequencer, and 151 bp paired-end reads were generated using the NovaSeq sequencing platform. We then conducted adapter trimming and followed quality control procedures as described previously (28). The reads were then mapped using STAR to build human GRCh37; the gene expression levels were quantified and normalized by HTSeq and DESeq2 as previously described (28). Negative binomial models in DESeq2 were used to conduct differential expression analysis. We used the skin biopsies obtained from our previous study to increase the sample size for the control samples (45). The data accession nos. are GSE154557 and GSE179162 (Gene Expression Omnibus [GEO]; https://www.ncbi.nlm.nih.gov/geo/). Data processing, including read alignment, gene quantification, and quality control, was conducted utilizing the 10x Genomics Cell Ranger software. Seurat was used for normalization, data integration, and clustering analysis (46). Clustered cells, including keratinocyte populations, were mapped to corresponding cell types by matching cell cluster gene signatures with the putative cell type–specific markers (28).

### Primary keratinocyte isolation.

Primary keratinocytes were isolated from the tails or wounds of mice as previously described (29). First, skin from tails and wounds was incubated in 1% trypsin for 1.5 hours and 2 hours, respectively, at 37°C. Following incubation, the epidermis from tails and wounds was peeled off, and epidermal cells (containing primarily keratinocytes) were agitated off into high-calcium (HiCa) medium (8% nonmixed FBS in EMEM). Next, primary keratinocytes from the tail were cultured in low-calcium (LoCa) media (8% mixed FBS in EMEM, at 0.05 mM final Ca^2+^ concentration) for 3 days at 37°C. Following isolation of primary keratinocytes at baseline (tail) and wounds, cells were harvested for RNA or fixed in 1% paraformaldehyde for ChIP.

### qPCR.

Before RNA extraction, snap-frozen wounds were homogenized as previously described (47). Once homogenized, wounds, keratinocytes, and isolated monocyte/MΦ were placed in TRIzol (Invitrogen). RNA was isolated using chloroform, isopropanol, and ethanol. iScript (Bio-Rad) was then used to reverse transcribe RNA to cDNA. Real-Time PCR was performed with 2× TaqMan PCR Mix or 2× SYBR Green mix via the 7500 Real-Time PCR System. Primers used were as follows: murine *Ifnk* (forward, 5′-TACGATAGGAGACGGCGTTTA-3′; reverse, 5′-ACTCCAAAGTTTTTATGGCTGGT-3′), *Il1b* (Mm00434228), *Tnfa* (Mm00443258), *Il12* (Mm00434165), human *IFNk* (forward, 5′-GTGGCTTGAGATCCTTATGG GT-3′; reverse, 5′-CAGATTTTGCCAGGTGACTCTT-3′), *Mll1* (Mm01179235), *Col1* (Mm00801666), *Vegfa* (Mm00437306), and *18s* (no. 4318839) as internal control (Applied Biosystems). All real-time PCR experiments were run on a 7500 Real-Time PCR System (Applied Biosystems). Data were examined in a relative quantification analysis to 18S (2^–ΔΔCt^). All samples were performed in triplicate.

### ChIP.

As previously described (48), cells were fixed in 1% paraformaldehyde and glycine. This was followed by lysis and sonication using a Bioruptor Pico (Diagenode) to generate 300–500 bp fragments. Samples were then incubated overnight with anti-H3K4me3 antibody (39159, Active Motif), anti-MLL1 antibody (61296, Active Motif), or isotype control (rabbit polyclonal IgG; ab171870, Abcam), followed by the addition of protein A or protein G Sepharose beads (Thermo Fisher Scientific). Beads were washed; then, DNA was eluted and purified using phenol/chloroform/isoamyl alcohol extraction. The DNA was then precipitated using ethanol. H3K4me3 deposition was measured by qPCR using 2× SYBR PCR mix (Invitrogen), and primers (forward, 5′-TCTCTGACACCTTTGCCTGA-3′; reverse, 5′-CCAGTTCTCTTCCCTTGCAC-3′) were designed to flank the *Ifnk* promoter using NCBI Primer-BLAST.

### Wound digestion for magnetic cell sorting and flow cytometry.

Wounds were digested as previously described (28). Briefly, wounds were minced and digested by incubating in a 20 U/mL DNase I (Sigma-Aldrich) and 50 mg/mL Liberase TM (Roche) solution for 30 minutes at 37°C. Following incubation, wound cell suspensions were filtered through a 100 μm filter to achieve single-cell suspension. Cells were then stained for flow cytometry or magnetic cell sorted for RNA isolation.

### Flow cytometry.

Single-cell suspensions were collected from wounds as described above, washed with PBS, and filtered into a 96-well plate for surface staining. Cells were then resuspended in Flow Buffer (PBS, FBS, NaN3, and HEPES buffer), and Fc-receptors were blocked with anti-CD16/32 (BioLegend) before surface staining. Monoclonal antibodies for surface staining included: anti-CD3 (BioLegend, 100237), anti-Ly6G (BioLegend, 127605), anti-CD11b (BioLegend, 101212), anti-CD45 (BioLegend, 1003115), and anti-Ly6C (BioLegend, 128035). All antibodies were used at 1:100 dilutions. Following surface staining, cells were washed twice with PBS and stained with BV 510 LIVE/DEAD fixable viability dye (Thermo Fisher Scientific). After viability dye staining, cells were washed twice with PBS; then, samples were acquired on a 3-Laser FACSCelesta flow cytometer (BD Biosciences). Data were analyzed using FlowJo software.

### Magnetic isolation of monocytes/MΦ.

After collecting single-cell suspensions from wounds, cells were incubated with biotin-labeled anti-CD3 (BioLegend, 100304), anti-CD19 (BioLegend, 115504), and anti-Ly6G (BioLegend, 127604), followed by incubation with streptavidin RapidSpheres (Stemcell Technologies, 19860A). Flow-through was then incubated with anti-CD11b and Dextran RapidSpheres (Stemcell Technologies, 18970A) to isolate the nonneutrophil, nonlymphocyte, and CD11b^+^ cells. Cells were saved in Trizol (Invitrogen) for qPCR analyses.

### Statistics.

All data were analyzed and graphed using GraphPad Prism software version 8. Data comparing differences between more than 2 groups were analyzed using 1-way ANOVA. Analysis between 2 groups was done using a 2-tailed Student’s *t* test for groups normally distributed. For data with unequal variances, Welch’s correction was applied. For nonnormally distributed data, a Mann-Whitney *U* test was performed. A *P* value less than 0.05 was considered significant.

### Study approval.

Mice were housed in the University of Michigan pathogen-free animal facility, and all protocols were approved by and in compliance with the guidelines established by the IACUC at the University of Michigan. Patient consent for collecting human wound tissue was exempt by the IRB because tissue was collected from discarded surgical material (protocol no. HUM00060733).

## Author contributions

KAG, SJW, AJ, and JMK designed the experiments. SJW, AD, AJ, COA, XX, RW, LCT, FMD, and WJM performed experiments. SJW, AD, AJ, COA, WJM, and JEG analyzed data. SJW and KAG prepared the manuscript. JMK, SLK, JEG, MXO, and LCT reviewed and edited the manuscript. SJW and KAG are the guarantors of this work and, as such, had full access to all the data in the study; they take responsibility for the integrity of the data and the accuracy of the data analysis.

## Supplementary Material

Supplemental data

## Version 1 03/31/2022

In-Press Preview

## Version 2 05/09/2022

Electronic publication

## References

[1] Alsabbagh MW (2015). Trends in prevalence, incidence and pharmacologic management of diabetes mellitus among seniors newly admitted to long-term care facilities in Saskatchewan between 2003 and 2011. Can J Diabetes.

[3] Parekh PI (1998). Reversal of diet-induced obesity and diabetes in C57BL/6J mice. Metabolism.

[4] Surwit RS (1998). Diet-induced changes in uncoupling proteins in obesity-prone and obesity-resistant strains of mice. Proc Natl Acad Sci U S A.

[5] Park SY (2005). Unraveling the temporal pattern of diet-induced insulin resistance in individual organs and cardiac dysfunction in C57BL/6 mice. Diabetes.

[6] Kimball AS (2019). The histone methyltransferase Setdb2 modulates macrophage phenotype and uric acid production in diabetic wound repair. Immunity.

[7] Kruidenier L (2012). A selective jumonji H3K27 demethylase inhibitor modulates the proinflammatory macrophage response. Nature.

[8] Martinez FO (2009). Alternative activation of macrophages: an immunologic functional perspective. Annu Rev Immunol.

[9] Martinez FO (2008). Macrophage activation and polarization. Front Biosci.

[10] Jiang Y (2020). Cytokinocytes: the diverse contribution of keratinocytes to immune responses in skin. JCI Insight.

[11] Porcheray F (2005). Macrophage activation switching: an asset for the resolution of inflammation. Clin Exp Immunol.

[12] Kimball A (2018). Ly6C ^Hi^ blood monocyte/macrophage drive chronic inflammation and impair wound healing in diabetes mellitus. Arterioscler Thromb Vasc Biol.

[13] Piipponen M (2020). The immune functions of keratinocytes in skin wound healing. Int J Mol Sci.

[14] Fujisawa H (1997). The expression and modulation of IFN-alpha and IFN-beta in human keratinocytes. J Interferon Cytokine Res.

[15] Stannard JN (2017). Lupus skin is primed for IL-6 inflammatory responses through a keratinocyte-mediated autocrine type I interferon loop. J Invest Dermatol.

[16] Sarkar MK (2018). Photosensitivity and type I IFN responses in cutaneous lupus are driven by epidermal-derived interferon kappa. Ann Rheum Dis.

[17] Kong J, Li YC (2002). Upregulation of interleukin-18 expression in mouse primary keratinocytes induced to differentiate by calcium. Arch Dermatol Res.

[18] Lan CC (2008). Hyperglycaemic conditions decrease cultured keratinocyte mobility: implications for impaired wound healing in patients with diabetes. Br J Dermatol.

[19] Song ZQ (2008). [Impact of advanced glycosylation end products-modified human serum albumin on migration of epidermal keratinocytes: an in vitro experiment]. Zhonghua Yi Xue Za Zhi.

[20] Werner S (1994). Induction of keratinocyte growth factor expression is reduced and delayed during wound healing in the genetically diabetic mouse. J Invest Dermatol.

[21] Galkowska H (2003). Expression of apoptosis- and cell cycle-related proteins in epidermis of venous leg and diabetic foot ulcers. Surgery.

[22] Li Y (2019). Interferon kappa is up-regulated in psoriasis and it up-regulates psoriasis-associated cytokines in vivo. Clin Cosmet Investig Dermatol.

[23] Gharaee-Kermani M (2022). IFN-κ is a rheostat for development of psoriasiform inflammation. J Invest Dermatol.

[24] Na J (2016). Histone H3K27 demethylase JMJD3 in cooperation with NF-κB regulates keratinocyte wound healing. J Invest Dermatol.

[25] Na J (2017). JMJD3 and NF-κB-dependent activation of Notch1 gene is required for keratinocyte migration during skin wound healing. Sci Rep.

[26] Jaenisch R, Bird A (2003). Epigenetic regulation of gene expression: how the genome integrates intrinsic and environmental signals. Nat Genet.

[27] Davis FM (2019). Histone methylation directs myeloid TLR4 expression and regulates wound healing following cutaneous tissue injury. J Immunol.

[28] Davis FM (2020). Epigenetic regulation of the PGE2 pathway modulates macrophage phenotype in normal and pathologic wound repair. JCI Insight.

[29] Lichti U (2008). Isolation and short-term culture of primary keratinocytes, hair follicle populations and dermal cells from newborn mice and keratinocytes from adult mice for in vitro analysis and for grafting to immunodeficient mice. Nat Protoc.

[30] Boniakowski AM (2019). SIRT3 regulates macrophage-mediated inflammation in diabetic wound repair. J Invest Dermatol.

[31] Wolf SJ (2021). Macrophage-mediated inflammation in diabetic wound repair. Semin Cell Dev Biol.

[32] Dal-Secco D (2015). A dynamic spectrum of monocytes arising from the in situ reprogramming of CCR2+ monocytes at a site of sterile injury. J Exp Med.

[33] Tartaglia LA (1995). Identification and expression cloning of a leptin receptor, OB-R. Cell.

[34] Robert I (2009). Matrix metalloproteinase-9 gene induction by a truncated oncogenic NF-kappaB2 protein involves the recruitment of MLL1 and MLL2 H3K4 histone methyltransferase complexes. Oncogene.

[35] Carson WFt (2017). The STAT4/MLL1 epigenetic axis regulates the antimicrobial functions of murine macrophages. J Immunol.

[36] Kimball AS (2017). The histone methyltransferase MLL1 directs macrophage-mediated inflammation in wound healing and is altered in a murine model of obesity and type 2 diabetes. Diabetes.

[37] Wang X (2012). MLL1, a H3K4 methyltransferase, regulates the TNFα-stimulated activation of genes downstream of NF-κB. J Cell Sci.

[38] Nardelli B (2002). Regulatory effect of IFN-kappa, a novel type I IFN, on cytokine production by cells of the innate immune system. J Immunol.

[39] Scarponi C (2006). Analysis of IFN-kappa expression in pathologic skin conditions: downregulation in psoriasis and atopic dermatitis. J Interferon Cytokine Res.

[40] LaFleur DW (2001). Interferon-kappa, a novel type I interferon expressed in human keratinocytes. J Biol Chem.

[41] Zou ML (2021). Fibroblasts: heterogeneous cells with potential in regenerative therapy for scarless wound healing. Front Cell Dev Biol.

[42] Bao P (2009). The role of vascular endothelial growth factor in wound healing. J Surg Res.

[43] Surwit RS (1995). Differential effects of fat and sucrose on the development of obesity and diabetes in C57BL/6J and A/J mice. Metabolism.

[44] Leiter EH (2009). Selecting the “right” mouse model for metabolic syndrome and type 2 diabetes research. Methods Mol Biol.

[45] Tsoi LC (2020). Progression of acute-to-chronic atopic dermatitis is associated with quantitative rather than qualitative changes in cytokine responses. J Allergy Clin Immunol.

[46] Butler A (2018). Integrating single-cell transcriptomic data across different conditions, technologies, and species. Nat Biotechnol.

[47] Wolf SJ (2019). Ultraviolet light induces increased T cell activation in lupus-prone mice via type I IFN-dependent inhibition of T regulatory cells. J Autoimmun.

[48] Ishii M (2009). Epigenetic regulation of the alternatively activated macrophage phenotype. Blood.

