# Supplementary material for: IFN-κ is critical for normal wound repair and is decreased in diabetic wounds
Source: JCI Insight. 2022 May 9;7(9):e152765. doi: 10.1172/jci.insight.152765 (PMC9090246; doi:10.1172/jci.insight.152765)
Supplement: Supplemental data [file jciinsight-7-152765-s008.pdf]

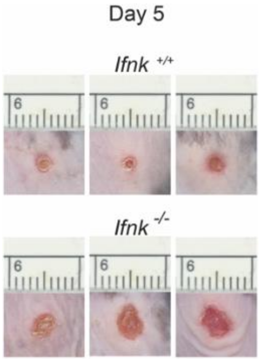

Supplemental Figure 1: Deletion of IFNκ impairs normal tissue repair. 4mm punch biopsy wounds were created on *Ifnk*$^{+/+}$ and *Ifnk* $^{-/-}$ mice (2 wounds per mouse,5 mice per group). Three representative photographs of the wounds were taken on day 5.

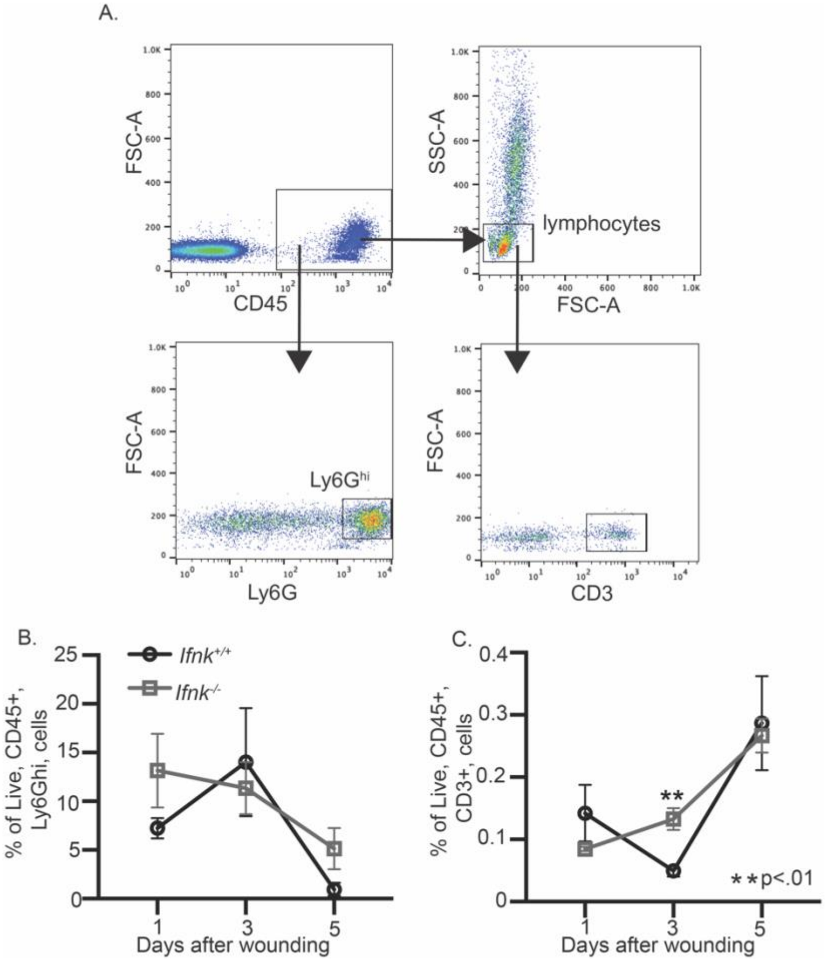

Supplemental Figure 2: IFNκ regulates T cells during normal tissue repair. (A). IFNκ KO and WT wound cell isolates were processed for flow cytometry using the following gating strategy selecting for neutrophils (live, CD45+, Ly6Ghi) or T cells (live, CD45+, CD3+). (B). Flow cytometry quantification of neutrophils in wounds (n = 4-6 per group). (C) Flow cytometry quantification of T cells in wounds (n = 4-6 per group). Data were analyzed for variances, and 2-tailed student t-test was performed. **p < 0.01. Data are presented as mean and SEM.

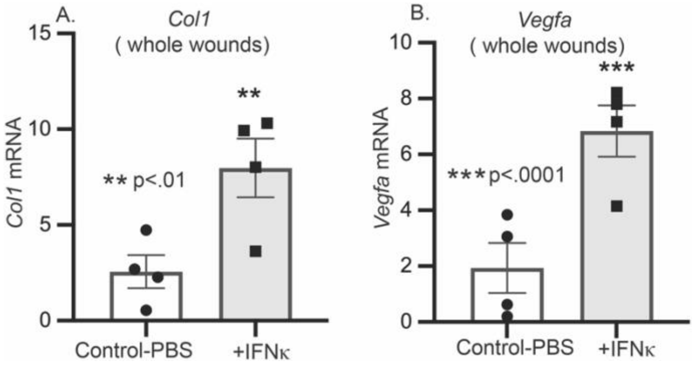

Supplemental Figure 3: IFNκ induces *Col1* and *Vegf* expression in wounds. (A and B) Wounds isolated from IFNκ and PBS control mice, n=4 per group. Gene expression of *Col1* and *Vegfa* was measured via qPCR. Data were analyzed for variances, and 2- tailed student t-test was performed. **p < 0.01 and ***p < 0.001. Data are presented as mean and SEM.

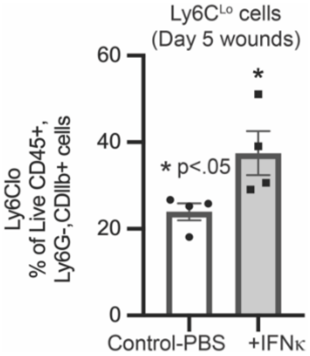

Supplemental Figure 4: Ly6Clo macrophage/monocyte increased following IFNκ rescue in DIO mice. (A and B) Wounds isolated from IFNκ and PBS control mice, n=4 per group. Flow cytometry quantification of Ly6Clo cells in wounds (n = 4 per group). *p < 0.05. Data are presented as mean and SEM.
